# Experiences of the First Year Implementation of a Nationwide School-Based Smoking Prevention Program in Korea

**DOI:** 10.3390/ijerph18063291

**Published:** 2021-03-22

**Authors:** Sookyung Kim, Seunghyun Yoo, Sung-il Cho, Hanna Jung, Yeaseul Yang

**Affiliations:** 1 College of Nursing, Yonsei University, Seoul 03722, Korea; sookyungkimm@gmail.com; 2 Graduate School of Public Health and Institute of Health and Environment, Seoul National University, Seoul 08826, Korea; scho@snu.ac.kr; 3 Institute of Health and Environment, Seoul National University, Seoul 08826, Korea; junghanna0324@gmail.com (H.J.); yys2863@snu.ac.kr (Y.Y.)

**Keywords:** smoking prevention, school health, program implementation, qualitative research

## Abstract

Encouraged by the Framework Convention on Tobacco Control, Korea has implemented a nationwide School-based Smoking Prevention Program (SSPP) to reduce the prevalence of youth smoking. This qualitative study explored the school contexts of launching the SSPP in Seoul, Korea. Five focus groups were studied with 29 lead teachers in charge of the SSPP. Thematic analysis reveals three key findings. First, while infrastructure was insufficiently prepared due to the abrupt implementation, lead teachers agreed on the purpose of the SSPP. However, they perceived the program as myopic in only targeting smoking students and spending the mandatory budgets as a burdensome task. Second, the SSPP increased experience-based activities, influenced smoking family members, and created a smoke-free school environment. Third, to ensure more effective implementation, school principals should support inducing staff engagement. The teachers also maintained that the SSPP must be institutionalized as part of regular curricula with standardized books. For a more meaningful impact, the SSPP needs instructors and counselors to support smoking cessation programs that reflect school contexts. The teachers urged tobacco prevention measures at community, policy, and society levels. This study provides insights into a nationwide approach to initiating school-based smoking prevention program to achieve a tobacco-free generation.

## 1. Introduction

Smoking results in about seven million deaths worldwide each year [1]. Between 1980 and 1990, South Korea (hereinafter, Korea) had the highest prevalence of male smokers in the world. In 1985, 71.1% of Korean men were smokers, as were 10.7% of the female population [2]. Smoking prevalence in Korea has gradually declined to reach 43.1% of men and 5.7% of women in 2014 [3]. However, the prevalence of daily smoking among Korean adolescents has increased from 3.9% in 2005 to 4.8% in 2014. Specifically, the prevalence of daily smoking among Korean adolescent males was 7.5% in 2014 and 3.6% for their female counterparts. The initial age of smoking in Korea remained consistent from 12.0 years in 2005 to 12.6 years in 2014 [4]. Before the implementation of school-based smoking prevention programs (hereinafter, SSPP), the smoking rate of the total adolescents was 9.2%, but from 2016, it has remained at approximately 6% [4].

The Korean government has taken steps to reduce adolescent smoking. The most significant was the implementation of SSPP in all elementary, middle, and high schools in the country since 2015. Prior to the SSPP, mandatory school health education in Korea was focused on the prevention of substance abuse overall including tobacco, alcohol, and drugs, which required smoking prevention education at least twice a year in elementary school and at least once a year in middle and high school [5]. Such smoking prevention education was typically provided by health teachers, who are registered nurses and are responsible for the health education and care of students. They are employed full-time in every school in Korea [6]. In an additional effort, the Smoking Cessation Leading School program, where 10% of all schools in Korea attempted to prevent or reduce tobacco use among adolescents, was implemented in 2013–2014 [7]. Korea Health Promotion Institute reported that because of this program, adolescent’s knowledge about smoking increased by 5.3%, and smokers who attempted to quit smoking increased by 13.7% in 2014 [8]. Since 2015, SSPP has been implemented to operate a more specific and comprehensive program than its predecessors at all schools across the country. The ultimate goal of the SSPP is to foster tobacco-free generations [9]. The SSPP constitutes a more intense and structured form of preventative education.

In terms of both policy and budget, the raising of the average tobacco price, which was 80.0% (about USD 2) on January 1st, 2015 [10], since the price has been frozen for the last 10 years, influenced the launching of SSPP. The implementation of the program in all the schools in Korea was wholly funded by the national budget. Prior to the SSPP, only 10% of the schools in Korea which implemented the Smoking Cessation Leading School program were provided with a budget [11].

Countries such as Australia, Singapore, and the United Kingdom implement tobacco prevention school education at a national level, emphasizing multi-pronged approaches, interactive delivery methods, and tobacco-free policies [12,13,14]. Article 12 of the Framework Convention Tobacco Control (FCTC) developed by the World Health Organization (WHO) emphasizes the need to improve awareness of smoking through education and communication [15]. In 2014, Article 12 had an average implementation rate of 70% among FCTC members [16]. Korea has been moving in the same direction to develop and release anti-smoking campaigns since 2014 [16], while in need of enhancing school-based smoking prevention education to achieve tobacco-free generations. Such enhancement is required particularly for context-based program implementation. Nationwide implementation of an intervention is exposed to several contextual determinants due to its wide range of coverage. Program implementation which takes into consideration the school’s socio-environmental context, supported by in-depth situation evaluation, is crucial for a successful program [17]. In a Danish study of smoking prevention at about 100 schools, school contextual factors such as administrative leadership and supportive school environment for program staff were more influential for successful program implementation than school size or the proportion of students with risky behaviors [18].

This study aimed to qualitatively explore the school contexts of SSPP after the first year of the nationwide program implementation in Korea in 2015. Since the actual content and implementation of the program are decided by the schools themselves, its research questions are: (1) How is SSPP being implemented at schools? and (2) What are the school contexts and how does this relate to the successful implementation of improvements in SSPP?

## 2. Materials and Methods

### 2.1. The Intervention

School education in Korea starts at the age of six. It consists of nine years of compulsory education at the time of the study (six years of elementary school and three years in middle school) [19], and three years in high school. The SSPP is required for all schools in Korea in either a basic or an intensive program. Participation in an intensive program is on a voluntary basis, while the default is to participate in a basic program. The intervention contents according to the different program types are displayed in Table 1 [7]. In 2015, 10% of Korean schools participated in an intensive program and received an annual budget support of USD 4450–9000 per school. The remaining 90% of elementary, middle, and high schools implemented the basic program with an approximate annual budget support of USD 1350–2650 per school (Table 1) [7]. SSPP guidelines (Table S1) recommend using Experience New Days (END) as a standardized textbook developed by the Korea Health Promotion Institute [7].

To examine the implementation of SSPP, this study focused on Seoul, the largest jurisdictional area of the Office of Education in Korea. In 2015, there were 599 elementary schools, 384 middle schools, 318 high schools, and 38 schools of other types (e.g., special schools) in Seoul for approximately 1.03 million students and 72,000 teachers [20]. The lead teacher of the SSPP is typically a health teacher or a discipline and safety department teacher [21].

### 2.2. Data Collection

Focus groups, a method of sharing and comparing thoughts and opinions among participants, was conducted for comprehensive understanding of experiences, perceptions, and beliefs [22]. We recruited and conducted four types of focus groups in 2016 divided according to program type (basic or intensive) and school type (elementary or middle, and high school) in order to gather the key opinions of each type. Purposive sampling recruited teachers who had overseen the SSPP for a year or more at elementary, middle, and high schools in Seoul. The recruitment was conducted in cooperation with the Seoul Metropolitan Office of Education, Seoul School Health Promotion Center, and Seoul Health Teachers Association. Participants were recruited from schools, agencies, and organizations of school education and school health by email, telephone, and cooperation documents. At the beginning of the recruitment, 24 people participated by contacting around 50 sites. Additionally, five more people participated by contacting teachers from a total of 21 schools that met the criteria for participation in the study through the snowballing method. Initial participants were predominantly health teachers responsible for most of the elementary school SSPP. Teachers responsible for SSPP were mainly in two positions (health teacher and teacher in a discipline and safety department) in middle and high schools. Therefore, two focus groups were formed according to each position in the basic program at middle and high schools. A total of 29 teachers participated in five focus groups, each comprising five to seven participants (Table 2).

Prior to the focus group discussions, the research team explained the purpose of the study and the focus groups process to the participants, including that they would be recorded. Written informed consent was obtained and participants were provided with a copy of the consent form. A short survey was administered to collect the basic sociodemographic information of the participants. Conducted between September and October 2016, the semi-structured focus groups lasted for 120–150 minutes per group. One researcher and more than one research assistant attended each focus group session: the researcher facilitated the focus group while the research assistants took notes. The major focus group questions included the planning process and contents of the SSPP that were implemented, use of the END textbook in the SSPP, implementation issues, evaluation methods, and the perceived effects of the SSPP. The focus groups concluded by reviewing the topics with the participants and asking whether they wished to clarify or expand on anything discussed during the focus group. All verbal data were audio-recorded, transcribed verbatim, and enhanced by field notes taken during data collection. The study protocol was approved by the Institutional Review Board of the corresponding author’s institution (IRB No. 1609/001-007 & E1610/003-015).

### 2.3. Data Analysis

Thematic analysis [23] was performed on the focus group data through an inductive analysis approach. We conducted open-coding of meaningful units related to the SSPP. Code matrix was created in an Excel program. After duplicated codes were integrated or deleted, similar meaningful codes were categorized and named codes which were considered to encompass the group of codes. Next, we performed another categorizing process to discover the themes by sorting the different codes into potential themes and collating the relevant coded data extracts within the identified themes. During the process, we continuously discussed the coding process, code structure, and constant comparison facilitating the reclassification and restructuring of the codes based on their conceptual relationships. We ended the theme search with a collection of candidate themes, and sub-themes, and extracted all of the data. By reviewing, the themes were held, combined, refined, and separated or discarded. Finally, contexts of SSPP were identified and organized into three main themes and eleven categories derived from 91 codes based on the data. Quotes were translated from Korean into English by S.K. and verified by S.Y. In order to ensure the trustworthiness of this study, the data collection and analysis process were based on the quality criteria of Guba and Lincoln [24]. For the credibility of the data, participants who could express the rich experience in SSPP were selected considering various characteristics such as age, position, school type, school size, and we read the transcripts repeatedly and analyzed them through an iterative process. For dependability of the data, analyzed results were inspected independently by two researchers. To improve the transferability of the research, the context in which the focus groups were studied, setting, recruiting methods, demographic characteristics, and the process of the focus groups were described in detail. To ensure confirmability, we asked about statements whose meaning was unclear, clarifying them during the focus groups, and analyzed results were shared and discussed with all of the research members.

## 3. Results

The demographic characteristics of participants are shown in Table 3. The school contexts of SSPP implementation are described under three main themes: (i) educationally important but technically insufficient, (ii) perceived impacts of the SSPP, and (iii) suggestions for more structured SSPP (Table 4).

### 3.1. Educationally Important But Technically Insufficient

#### 3.1.1. Agreement with the SSSP’s Purpose

Most lead teachers—that is, those in charge of the SSPP at their respective schools—expected the non-smoking students of SSPP to have a lower likelihood of smoking. While lead teachers believed that SSPP implementation needs improvement, the majority agreed on its expected effect. They strongly agreed with the program’s focus on smoking prevention rather than cessation. Lead teachers anticipated that the smoking rate of Korea will decrease because of current education. Lead teachers at elementary schools were particularly positive toward the SSPP’s purpose, believing that smoking prevention education at elementary schools will effectively prevent the children from smoking by using diverse educational activities. This optimism is reflected in the following participant’s statement:

*I did not expect much for the program. … Now I think the program has preventive effects on non-smoking students at least by informing them about the consequences of smoking with audio-visual materials. I came to think the program has some effects after hearing girls saying, “I won’t smoke.” I think the program has a possibility for smoking prevention.*

*(D-5)*

#### 3.1.2. Insufficient Preparation of Infrastructure Due to Abrupt Implementation

Schools were informed in a top-down manner about the Korean government’s decision to implement the SSPP in all schools nationwide using funds from increased tobacco taxes. The SSPP requires substantial efforts for the school to operate and manage, and lead teachers felt frustrated in the way such a school health program was announced and initiated unilaterally. There was also antipathy toward the program regarded by some as a justification for a tax increase. Moreover, health was an optional subject at middle and high schools, so it was not included in the curricula in many schools. Therefore, it was challenging for some schools to spare class time and space for smoking prevention education that is not part of a regular curriculum. Abrupt mandatory implementation made the SSPP difficult for lead teachers to administer.

Lead teachers also noted that some of the SSPP contents were similar to those of already available public programs offered by the District Office of Education, Seoul School Health Promotion Center, and Community Health Center. Some schools were informed about such public resources available for low or no cost only after employing puppet shows and musicals in their SSPP by hiring services [to spend the] school budget.

*It’s too much for schools to do everything. It would be efficient to divide and clarify roles. For smoking prevention, schools focus on its fundamental role of education, Community Health Centers host events, and the Office of Education leads campaigns.*

*(A-2)*

A shared opinion was that implementation support for the SSPP lacked contextual understanding of school-based programs. While schools set annual plans at the beginning of the calendar year before an academic year begins in March in Korea, a list of public resources for the SSPP became available in the middle of the year. Some event materials sent to schools from a public agency without prior consultation were unused and discarded.

#### 3.1.3. Narrow Perspective of the SSPP Education for Parents and Teachers

Most lead teachers tended to consider that the SSPP education for parents was designed for those with smoking children and adolescents. A few lead teachers believed that parents of smoking students should be involved in the SSPP activities in school. However, parental involvement was one of the most difficult tasks for the teachers particularly in the daytime on weekdays, and most schools chose to distribute printed materials instead. Already existing school events such as festivals and athletic competitions were considered as a great opportunity to link to the SSPP to connect with parents.

*The hardest thing is parent education. In the area where my school is located, most parents are working. When I host an education session for parents at school, less than ten parents participate. And those participants are mostly the parents of well behaving students.*

*(D-5)*

Lead teachers also thought that the SSPP for teachers was only for teachers who smoke. Smoking teachers tended to feel opposed to the SSPP created and financed by the tobacco tax increase, which made it difficult for the lead teachers to intervene. Another point was that many elementary teachers are women in Korea where the prevalence of female smoking is low. For those lead teachers in elementary schools, it was difficult to apply the SSPP to their teacher colleagues who thought the SSPP was intended to identify smokers and coerce them to quit smoking.

#### 3.1.4. A Burdensome Task of Spending SSPP Budget

In contrast with its predecessors, the SSPP consists of multiple elements to implement and a mandatory annual budget to spend. For some of the educational activities on smoking prevention could be provided without a budget as they had been prior to the SSPP, spending the mandatory SSPP budget took priority over developing and implementing a quality program. Lead teachers felt pressured to spend the budget, otherwise they had to write a case report on the budget that was unused and returned. An unused program budget could also mean that a teacher did not do the work.

*The government demonstrates that tobacco price was raised for the health of the people and part of the raised tax is given to schools for smoking prevention. But teachers only agonize over how they can use all of the given budget.*

*(D-2)*

### 3.2. Perceived Impacts of the SSPP

Although some challenges were experienced as described in the first theme, the teachers noticed the unique characteristics and impacts of the SSPP.

#### 3.2.1. Increased Number of Experience-Based Activities on Smoking Prevention

A noticeable distinction between the SSPP and its predecessors was the its orientation toward experience-based learning in smoking prevention. The teachers evaluated that experiential learning in the SSPP was considered highly satisfactory by both students and teachers. Students were interested in and enjoyed the exhibits and experience provided at SSPP booths set up in schools and hands-on activities to make campaign products. The SSPP booths offered activities for students to engage in lung capacity measurement, visual displays of health consequences of smoking, and fingerprinting of a promise not to smoke in the future. Lead teachers appreciated that students were able to talk about smoking with one another while viewing the exhibition pieces. For a smoking prevention campaign, which is essential to the intensive type SSPP, students designed picket signs or campaign t-shirts. Due to the difficulty in securing class hours, students’ writing and posters on smoking prevention were displayed in schools.

*Of 17 health education sessions I had per class per semester, I let students make campaign items for one class. Then the students and I went to the subway station next to the school to do a 30-minute smoking prevention campaign.*

*(B-5)*

Schools that adopted the intensive SSPP type held a smoking prevention family camp involving both students and their parents. In middle and high schools, a safety map of the school area was created using a mobile phone application. Some schools participated in events hosted by the District Office of Education and Community Health Center that can be related to the SSPP.

*Our school did a painting on the wall at the request of the District Office of Education. The drawing was related to smoking prevention and done in an underground parking lot that had been messy. A lot of children were excited to have “a wonderful mural in our school.”*

*(A-3)*

#### 3.2.2. Positive Influence on Smoking Family Members

Most of the lead teachers—regardless of school and program type—acknowledged that the SSPP has a strong influence over family members who smoke, particularly in encouraging fathers to stop smoking. The concern of students regarding the health of their family and fathers during the smoking prevention class appears to have had a significant positive influence on the whole family. Indeed, a health teacher responsible for an intensive program at an elementary school reported that students had told her that “my dad said he is going to quit smoking” or “my father promised that he would smoke outside of the house from now on.” This effect on the family appears to be more significant in elementary schools than in middle and high schools.

#### 3.2.3. Creation of a Smoke-Free School Environment

The SSPP influenced enactment or amendment of smoking-related policies in schools specific to their situation. Schools employed penal policies for the students who smoke in school and created a norm for smoking prevention and a smoke-free environment.

*We could not go the toilets in our school due to cigarette smoke, just about three years ago. Now we are known as a school with no cigarette smell and smoke. In the change process several students had to be expelled for their smoking. Being next to smoking students is regarded as being smokers now. Since students often smoke because of their smoking friends, we focus on the importance of refusing, emphasizing that “If you cannot refuse, you are a smoker.” The students followed the atmosphere of the school.*

*(D-5)*

Although most participants were skeptical about implementing a smoking prevention program outside the school and influencing their community with the SSPP, there was an example that showed a potential for influencing the community illustrated in the quote below.

*My [middle] school is located together with a high school and a university. Our students go to school passing a smoking area in front of the university. Members of a school club ran a campaign once each semester last year to emphasize the need for a non-smoking area. After a while, schools brought up the problem officially and decided to close [the smoking area].*

*(E-4)*

### 3.3. Suggestions for a More Structured SSPP

#### 3.3.1. School Principals’ Support for Staff Engagement

Most significant for SSPP’s success was the positive attitude of principals to create the ideal school climate for smoking prevention. Principals’ support laid the foundation for SSPP implementation overall, creating a legitimate atmosphere for implementing the SSPP, fostering broader engagement of school staff, and influencing an active, effective operation of the SSPP committee. Training of principals is considered as a strategy for encouraging principals’ support to provide a comfortable climate for the SSPP in school.

*The principal of my school last year was a strong supporter of smoking prevention. A smoking cessation campaign was launched with the declarant of smoking prevention in school. The atmosphere has been a little different since a new principal came this year. He told me “don’t do anything related to smoking...”*

*(A-3)*

*The principal of my school, a smoker for 20 years, suggested [to] smoking teachers, “Let’s quit together.” A couple of staff members stopped smoking and the principal actively supported the smoking prevention program, so the other teachers cooperated as well.*

*(E-3)*

#### 3.3.2. Enhancing the Standard Textbook in Institutionalized Curricula

Lead teachers reported that the standard textbook for the SSPP, END, was not used widely in both basic and intensive programs because it contains excessive writing activities and assignments while lacking contents relevant to student-specific characteristics such as gender, school year, school type, and the health impacts of electronic cigarettes (e-cigarettes). The teachers requested SSPP textbooks and curricula specific to school level and even school years particularly for elementary school covering six years of student development. It was also emphasized that health classes for smoking prevention should be institutionalized as part of regular curricula.

*[The standard textbook] should be developed according to the target students. The current textbook is not usable for teens in general. Wouldn’t it be more helpful if there are textbooks for girls, boys, seniors in middle schools, and freshmen in high schools, for example?*

*(C-6)*

#### 3.3.3. Enhancing the Program Components for Smoking Students

Implementing a smoking cessation program for students was a demanding task for lead teachers. Smoking students tended to have underlying problems of low self-esteem and family issues, and the lead teachers needed professional advice and assistance from specialists to help these students. Meanwhile, the teachers evaluated that a school-based smoking cessation program for students can be effective with the following characteristics: a close rapport between teachers and smoking students; experience-based programs offered on a regular basis; and contents on self-esteem.

*It is important for a smoking cessation program to help students want to come to the program voluntarily. If there is no rapport [with the teacher], the students may pretend to have quit smoking in front of teachers but might not have actually quit.*

*(D-1)*

*[The] smoking cessation program is the most difficult to do. It is not easy to arrange time for it in school [to begin with]. I ran a program for five smoking students last year, which felt like teaching a hundred students. I burned out when the program was over.*

*(E-4)*

#### 3.3.4. Urging Tobacco Preventive Measures at Community, Policy, and Society Levels

Recognizing the influence of community and society on smoking in terms of tobacco sales, tolerance to tobacco, and media portrayal of tobacco products and smoking to name a few, the teachers noted that the prevention of youth smoking should include tobacco control efforts at community, policy, and social environment levels. However, they defined the SSPP as an education program for students within schools. Despite the recognized need for multilevel efforts to prevent youth smoking, the teachers, particularly those of basic SSPP, were not enthusiastic about extending the SSPP beyond schools. Although a teacher of intensive SSPP reported a positive experience of stopping tobacco sales to students at nearby stores by campaigning to ban tobacco sales to minors and reporting a case to the police, most teachers rather expected more preventive measures to be implemented at policy and society levels besides the SSPP.

## 4. Discussion

This study provides evidence for the school contexts of initiating a national smoking prevention project at the school level. In particular, the lead teachers recognized SSPP as important educationally, but they felt technically insufficient by the abrupt initiation of top-down approaches. Despite the lack of preparation for implementation, perceived impacts of SSPP resulted in increased experience-based activities, positive effects on family members, and creating smoke-free school environments. It was emphasized that the SSPP should be more structured in terms of principals’ support, enhancing the standard textbook in the institutionalized curriculum, and enhancing the program components for smoking students, and tobacco preventive measures at community, policy, and society levels. Based on these results, the SSPP is meaningful because it can be adapted when initiating large-scale school-based smoking prevention projects in other countries.

The principal’s leadership is essential so that the school staffs create a comfortable climate for the SSPP, which can allow many school staffs to engage in the operation of the SSPP [17,18]. A principal’s positive attitude toward the program is consistent with previous research that distributes work fairly and encourages teamwork [25]. In order for the principal to play this key role, training for principals would help them improve their understanding about the purpose of the SSPP through educating and providing specific information at the provincial or the national levels.

Our results showed that participants fully agreed that an SSPP program is effective in slowing the onset of smoking for non-smoking students and reduces the amount of tobacco usage among smoking students [26,27]. Nevertheless, almost all participants prioritized spending the SSPP budget rather than applying an effective approach and expressed difficulty in implementing programs for parents and teachers. Under the circumstances the actual contents of the program are decided by each school, and the lead teacher’s understanding and attitude toward SSPP is crucial. Therefore, lead teachers should be informed about the need for multilevel efforts to prevent youth smoking, the relationship between policy and the SSPP, and the goal of the SSPP at the nationwide initiatives: it is not only intended to promote smoking cessation, but also to encourage nonsmokers to remain committed against it [28]. A detailed program manual needs to be provided with step-by-step instructions and examples that can be applied to diverse school situations. For instance, indirect participation for parents through home-based activities such as creating educational materials and talking about a tobacco-free home could be included in the manual. This would enhance the meaning of a tobacco-free generation in terms of prevention, the reason why SSPP is so important, and the role of the teachers in the process.

Cooperation and coordination between institutions is considered at the stage of planning the SSPP. In this study, many institutional supports overlapped with SSPP and were inefficient for both the implementation and attainment of the particular SSPP goals of the individual schools. According to the WHO’s Global Standard for Health Promoting Schools, the role of each institution should be clearly identified in order to use resources and implement SSPP effectively [29]. Thus, diverse institutions must cooperate without overlapping the role of SSPP. When linking community resources and schools, it is necessary to check whether the process is suitable for the school system and its contexts. For example, unlike the fiscal year of administrative institutions, every school year starts in March and ends in February in Korea. The academic calendar should be considered when using out-of-school resources without a school for the SSPP.

These results were consistent with the results of a previous study that experience-based activities are effective in improving the knowledge and attitudes toward smoking [30]. To maximize the effectiveness of SSPP, multi-level tobacco control strategies should be implemented together as our results highlighted. The life of students does not stay only in the classroom. In everyday life, they see people who smoke, are exposed to tobacco marketing, and use stores selling tobacco. In particular, SSPP should be promoted as part of a comprehensive tobacco control policy. Three years after the SSPP was launched, the standard book was newly developed, and smoking prevention instructors and cessation counselors were trained and dispatched to the required schools [21,31,32]. In Seoul city, where this study was conducted, the youth smoking rate decreased from 7.0% in 2015 to 5.8% one year after the SSPP was implemented, and the smoking rate was maintained as low as 5.7% in 2019 [4]. Seoul is a large city, which is a significant economic, political, and cultural center for a country or region [33]. Compared to rural areas, a large city generally has abundant available resources, accessible services, and convenient transportation to operate the health promotional program. Therefore, it is meaningful to use the findings as evidence to adopt smoking prevention programs to large cities, and it is suggested that further study should be conducted to explore contexts for enhancing SSPP infrastructure in small cities and rural areas. Meanwhile, it is still necessary that smoking prevention education is included in the formal curriculum. New issues have arisen such as the increasing use of diverse tobacco products [34] and the lack of impacts on the smoking rate of female adolescents who exposed to many smoking programs in Korea [35]. In the 2020 guidelines for school smoking prevention program, the topic of the new tobacco products’ understanding was included for training of smoking cessation professionals [36]. Future education for adolescents should provide a specific topic on the issue about heated tobacco use due to its prevalence Korea. To create tobacco-free generations, the SSPP should be continuously revised to conform to the tobacco industry and policies of each country.

Although this study deals with the cases of smoking prevention programs in Seoul among Korea, SSPP is conducted in all schools nationwide. The prior study about the US national diabetes prevention program stated that the overall weight loss effects were achieved by increasing the duration and intensity of the session based on the national project [37]. The SSPP is a national-level program with increased class time supported by policy and budget. A future study is warranted to investigate the program’s impact on smoking prevention among children and adolescents in school. Meanwhile, in a study on preventive programs in Australia, it was found that the influencing factors of high evaluation quality were sufficient health promotion budget and national level prevention program operation [38]. The SSPP is funded and operated at a national level. Therefore, a further study is proposed to identify the impacts of smoking prevention, since it is high quality of intervention.

Although 76% of the participants were female, about 69% of teachers are female, as are 99% of the health teachers—the latter of whom tend to be responsible for SSPP in Korea [5]. Participants comprised current non-smokers. Therefore, the perspectives of teachers who smoke have not been reflected. Another limitation is related to the division of this research between four type of focus groups: basic elementary school, basic middle and high school, intensive elementary school, and intensive middle and high school. This may have resulted in a focus on how the SSPP programs were implemented overall rather than for each group specifically. Despite the limitations of this study, it has contributed by laying the foundation for the successful implementation of SSPP on a national level. The findings of this study help in formulating tactics for the international tobacco control community by taking into consideration the school context when a wide range of SSPP and other initiatives were sought to be implemented. Some of the tactics include making the principals play a key role, strengthening an institutionalized curriculum, and preparing effective measures at the community and social level.

## 5. Conclusions

This study contributes to the improvement of SSPP by generating strategies based on the exploration of SSPP implementation using the experiences of teachers involved. The theme of “educationally important but technically insufficient” results from insufficient preparation due to the abrupt initiation of the program, including a narrow perspective of the SSPP education for parents and teachers. Nonetheless, the results of this study indicate that the SSPP has a positive impact on reducing smoking rates. To improve the SSPP, this study suggests the creation of supportive environments through the positive attitudes of school principals, enhancing the standard textbook in institutionalized curricula, providing further support to teachers, and demarcating the roles of public institutions to prevent overlap. In addition, this study suggests the development of methods for educating parents and smoking teachers, as well as a smoking cessation program aimed at smoking family members. More research needs to be conducted among non-smoking students, smoking students who participate in SSPP, as well as the staffs of public institutions from a multilevel perspective.

## Figures and Tables

**Table 1:** Intervention characteristics according to program types.

		Basic Program	Intensive Program
**Contents**	Building an operational structure for the program in the school	R	R
	Providing smoking prevention education for students, school staff, and parents	R	R
	Organizing smoking prevention activities besides class sessions such as anti-smoking campaigns	R	R
	SSPP training for teachers	R	R
	Planning environmental analysis – needs assessment	O	R
	Operating the smoking cessation program for adolescents	O	R
	Developing and implementing an additional component of their own	N/A	R
	Cooperation with community	O	O
**Budgets**		USD 1350–2650 per school	USD 4450–9000 per school

R = required, O = optional, N/A = not applicable, SSPP = School-based Smoking Prevention Program.

**Table 2:** Composition of focus groups.

	Elementary School	Middle and High School
Basic program	Group A (*n* = 7)	Group C (*n* = 6) Group D (*n* = 5)
Intensive program	Group B (*n* = 5)	Group E (*n* = 6)

**Table 3:** Characteristics of study participants (*N* = 29).

No.	School Type	Number of Students	Characteristics	Program Type	Position	Gender	Age
A-1	Elementary	580	Coed school	Basic	Health teacher	F	43
A-2	Elementary	546	Coed school	Basic	Health teacher	F	37
A-3	Elementary	84	Coed school	Basic	Health teacher	F	37
A-4	Elementary	1500	Coed school	Basic	Health teacher	F	44
A-5	Elementary	350	Coed school	Basic	Health teacher	F	49
A-6	Elementary	381	Coed school	Basic	Health teacher	F	54
A-7	Elementary	990	Coed school	Basic	Health teacher	F	48
B-1	Elementary	500	Coed school	Intensive	Health teacher	F	48
B-2	Elementary	680	Coed school	Intensive	Health teacher	F	59
B-3	Elementary	800	Coed school	Intensive	Health teacher	F	47
B-4	Elementary	289	Coed school	Intensive	Health teacher	F	48
B-5	Elementary	800	Coed school	Intensive	Health teacher	F	57
C-1	Middle	750	Coed school	Basic	Health teacher	F	29
C-2	Middle	500	Coed school	Basic	Health teacher	F	52
C-3	Middle	410	Coed school	Basic	Health teacher	F	36
C-4	High	370	Vocational and girls school	Basic	Health teacher	F	47
C-5	Middle	350	Boys school	Basic	Health teacher	F	52
C-6	High	1300	Academic and Boys school	Basic	Health teacher	F	48
D-1	Middle	502	Coed school	Basic	Director of Discipline and Safety Division	M	42
D-2	Middle	560	Girls school	Basic	Director of Discipline and Safety Division	M	52
D-3	High	620	Academic and girls school	Basic	Director of Discipline and Safety Division	M	47
D-4	High	900	Academic and boys school	Basic	Health teacher	F	36
D-5	High	900	Vocational and coed school	Basic	Director of Discipline and Safety Division	M	57
E-1	Middle	850	Coed school	Intensive	Director of Discipline and Safety Division	M	57
E-2	Middle	1058	Coed school	Intensive	Director of Discipline and Safety Division	M	57
E-3	High	300	Vocational and coed school	Intensive	Health teacher	F	29
E-4	Middle	500	Girls school	Intensive	Health teacher	F	49
E-5	Middle	600	Coed school	Intensive	Director of Discipline and Safety Division	M	53
E-6	High	354	Vocational and girls school	Intensive	Health teacher	F	49

**Table 4:** Themes and categories from focus groups with School-based Smoking Prevention Program (SSPP) lead teachers.

Themes	Categories
**Educationally important but technically insufficient**	Agree with the SSPP’s purpose
	Insufficient preparation of infrastructure due to abrupt implementation
	Narrow perspective of the SSPP education for parents and teachers
	A burdensome task of spending SSPP budget
**Perceived impacts of the SSPP**	Increased number of experience-based activities on smoking prevention
	Positive influence on smoking family members
	Creation of a smoke-free school environment
**Suggestions for a more structured SSPP**	School principals’ support for staff engagement
	Enhancing the standard textbook in institutionalized curriculum
	Enhancing the program components for smoking students
	Urging tobacco preventive measures at community, policy, and society levels

## Data Availability

The data presented in this study are available on request from the corresponding author. The data are not publicly available to protect confidentiality of the research participants.

## Supplementary Materials

The following are available online at https://www.mdpi.com/1660-4601/18/6/3291/s1, Table S1: explanation of the SSPP.
