# Supplementary material for: Experiences of the First Year Implementation of a Nationwide School-Based Smoking Prevention Program in Korea
Source: Int J Environ Res Public Health. 2021 Mar 22;18(6):3291. doi: 10.3390/ijerph18063291 (PMC8005052; doi:10.3390/ijerph18063291)
Supplement: Supplementary file 1 [file ijerph-18-03291-s001.pdf]

Table S1. Explanation of the SSPP.

Role of school	• Selecting the teacher in charge of the SSPP.<br>• Organizing of a school committee involved with the SSPP.
Role of lead teacher	• Planning the SSPP based on the smoking survey and analysis of basic data.<br>• Submitting the SSPP plan via the online system.<br>• Implementing the planned SSPP.<br>• Self-evaluating the SSPP after its implementation.<br>• Submitting the SSPP self-evaluation via the online system.
Scope of SSPP	• Providing education for students, school staff, and parents.<br>• Forming a cooperation that actively utilizes parents, public health centers, and other relevant institutions to effectively achieve the purpose of the SSPP.<br>• Promoting smoking prevention activities linked to the extant curriculum.<br>• Linking with local public health centers and other relevant institutions to manage student smokers.<br>• Establishing school regulations for both students and staff in order to create a desirable environment.<br>• Participating in teacher training programs to strengthen their capacity to execute the SSPP effectively.

Abbreviation: SSPP = School-based Smoking Prevention Program.
